# Failure of intertrochanteric nailing due to distal nail jamming

**DOI:** 10.1007/s10195-012-0183-1

**Published:** 2012-02-24

**Authors:** Pietro Maniscalco, Fabrizio Rivera, Jacopo D’Ascola, Emmanuel Olivier Del Vecchio

**Affiliations:** 1 Department of Orthopaedic Surgery, Azienda Ospedaliera Piacenza, Via Taverna 49, Piacenza, Italy; 2 Department of Orthopaedic Surgery, Ospedale SS. Annunziata, Via Ospedali 14, Savigliano (CN), Italy

**Keywords:** Intramedullary nail, Complication, Femur, Fracture

## Abstract

Nail impingement against the anterior femoral cortex during nail insertion, or anterior cortex penetration, has been described in the literature as a worrying complication. We describe a previously unreported surgical failure due to a compromised dynamic distal locking caused by distal jamming of the nail. An 80-year-old male suffered a closed right intertrochanteric femoral fracture. Due to the presence of a long medial fragment, a 240 mm long titanium trochanteric nail was chosen to stabilize the fracture. Dynamic distal locking was performed by placing the distal screw at the inferior rim of the elliptical locking hole to allow compression of the fracture site during weight-bearing. Six-month X-ray follow-up revealed a broken nail and nonunion of the fracture due to failed dynamization of the distal locking screw. The nail was removed and replaced by a total arthroplasty. Due to the femoral anterior bow of the shaft, anterior cortical impingement of the distal tip of a nail may result in the failure of the nail to slide within the diaphyseal canal when using a medium-length nail preventing compression of the fracture. Dynamic distal locking can be ineffective if the ability of the distal nail to slide within the diaphyseal canal is hindered. This type of scenario can represent an opportunity for anterior nail impingement. Distal jamming of the nail can thus compromise dynamic compression at the fracture site during loading, thus inducing nonunion of the fracture, and leading to breakage of the osteosynthesis device. For these reasons, caution is recommended when using medium-length trochanteric nails for unstable trochanteric fractures.

## Introduction

Most intertrochanteric hip fractures can be treated successfully with internal fixation [1, 2]. Nonunion of intertrochanteric hip fracture is a relatively rare occurrence, with a reported incidence of 1–5% [3–5]. In order to decrease the incidence of failure, several variations of intramedullary nails have been devised. Nevertheless, the newer nail designs and materials can still result in complications such as cut-out of the implant [6], fracture of the femoral shaft distal to the tip of the implant [7], or medial migration of the implant [8]. The 1-year mortality after hip fracture can be as high as 20–30% [9].

We present a rare case of nonunion of an intertrochanteric fracture due to the failure of dynamic distal nail locking, as caused by distal jamming of the tip of the nail against the anterior cortex. A surgical failure due to distal jamming has never been described in the literature before.

## Case report

**Fig. 1:**
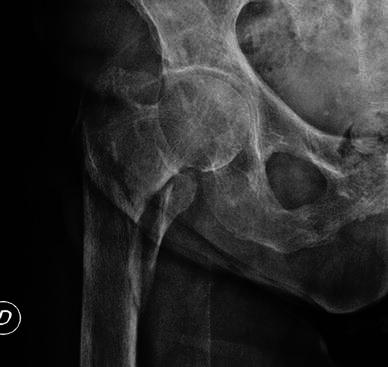
Preoperative anteroposterior radiograph shows an AO-OTA 31-A2.2 hip fracture

**Fig. 2:**
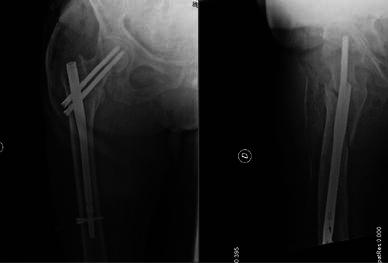
Postoperative radiographs show a medium-sized nail with distal dynamic locking. On the lateral view, the entry site appears to be correct (at the tip of the greater trochanter). Note that the bowing is not that significant

**Fig. 3:**
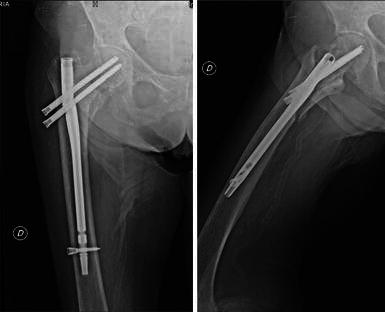
Six-month follow-up radiographs show nonunion of the intertrochanteric fracture and breakage of the nail. Note the sliding of the cephalic screws and the failed sliding of the nail around the distal screw. On the lateral view, the tip of the nail is abutting the anterior cortex

**Fig. 4:**
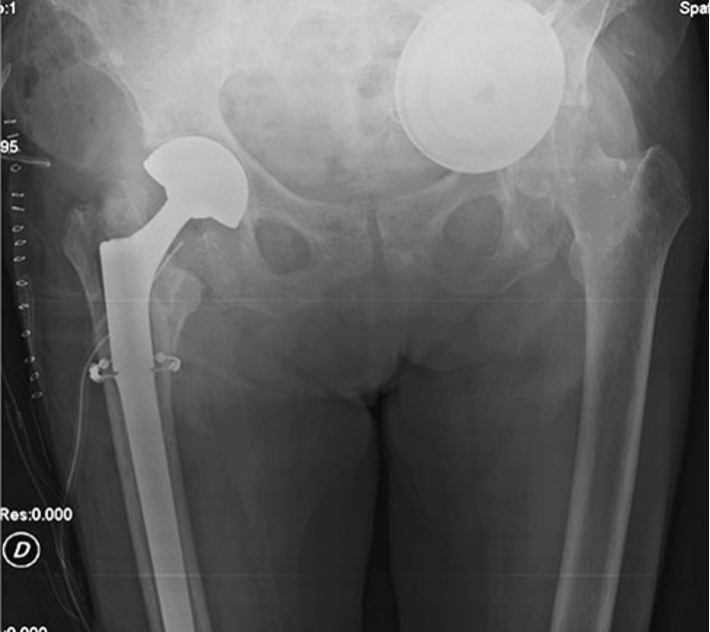
Postoperative radiograph after total hip arthroplasty

An 80-year-old male, 166 cm tall and weighing 56 kg, suffered a closed right intertrochanteric femoral fracture. X-rays in the emergency room revealed an unstable intertrochanteric fracture with more than two intermediate fragments (AO-OTA 31-A2.2 hip fracture) [10] (Fig. 1). Due to the presence of a long medial fragment, a 240 mm long titanium trochanteric nail (Endovis, Citieffe, Bologna, Italy) was chosen to stabilize the fracture (Fig. 2). Dynamic distal locking was performed by placing the distal screw at the inferior rim of the elliptical hole to allow compression on the fracture site during weight-bearing. Partial weight-bearing was allowed after 15 days. Postoperative X-rays at 1 month revealed nonunion of the fracture. The patient underwent monthly clinical and radiographical follow-up. Groin pain during walking and limping persisted during the following months. After 6 months, the patient had severe groin pain and some distal anterior thigh pain. X-rays revealed breakage of the nail and nonunion of the fracture due to failed proximal sliding of the distal screw within the distal elliptical locking hole (Fig. 3). During surgery, atrophic nonunion of the trochanteric fracture was observed. The nail was removed and replaced with a total hip arthroplasty combined with metallic cerclage around the distal fragment (Fig. 4). Two months after the hip replacement, the patient reported the disappearance of his groin pain.

The patient gave his informed consent prior to being included in this study.

## Discussion

Complications due to nail impingement with the anterior cortex during nail insertion, a difficulty encountered during nailing or anterior cortex penetration, have been well described in the literature [11–14].

What must be taken into consideration is the effect that the radius of curvature of the nail will have on the femur. Anthropologic studies have shown that the average radius of curvature of the human femur is 120 (±36) cm, with a range of 53–326 cm [11]. The radius of curvature does not differ significantly between genders. On the contrary, cortical and medullary bowing is strongly correlated with age, since the anterior bow in older people is greater than it is in young people, especially in women [15]. The radii of curvature of some commercially available nails may be greater. Intramedullary nails used for femoral fractures proximal to the anterior bow are at higher risk for distal anterior cortical penetration because of the mismatch in the radius of curvature between the nail and the femur [16]. For these reasons, in old ladies with short stature, a radiological evaluation that includes the full length of the femur should form part of the routine procedure before nailing is recommended [17]. As a result of this evaluation, patients with excessive bowing should possibly be treated with a dynamic hip screw instead [18].

Several complications related to distal tip nail impingement with femoral cortical bowing are described. In their series of unstable trochanteric and subtrochanteric fractures treated with proximal femoral nails, Menezes et al. [16] reported 1 case of a secondary fracture at the distal end of the nail. Ostrum et al. [12] described 3 cases of penetration of the distal femoral anterior cortex, stressing that this complication can occur with any technique or implant. Hwang et al. [17] reported 4 cases of technical difficulties related to the mismatch between the curvature of the nail and femoral shaft.

Full-length trochanteric nails should also be used in subtrochanteric fractures, rather than short trochanteric nails [19]. Advantages of a short intertrochanteric nail include a lower cost and the ability to insert distal locking screws using a targeting jig. Disadvantages include the possibility of fracture below the implant (stress riser effect) and the fact that a short nail does not protect the remaining femur in a patient with a history of falling and probable osteoporosis. The advantages of a full-length nail include the increased protection of the remaining femur. Disadvantages include increased cost over short nails, the need for free hand locking, and the mismatch of the anterior bow of the nail compared to the bow of the femur [20]. No available studies in the literature have considered the efficacy of short versus long trochanteric nails.

Moreover, the most recent generations of trochanteric nails offer multiple lengths of nails. The preoperative choice of length depends on the distal extent of the fracture. A medium-length nail allows easier insertion than a long nail and distal locking in combination with a targeting jig [21].

Another aspect of the latest generation of trochanteric nails is the possibility of dynamic distal locking (distal elliptical hole). Controlling fracture impaction through axial telescoping and rotational stability is essential in unstable proximal femoral fractures [22–24]. These factors allow direct contact between the fracture fragments, and promote healing, while decreasing the moment arm and consequent stresses on the implant. Compression at the fracture occurs during the healing process, under fracture loading.

Failure in our case was due to distal jamming of the nail into the anterior femoral cortex. Jamming prevented the distal sliding of the nail over the distal locking screw placed at the inferior rim of the elliptical distal hole. Thus, dynamic locking was ineffective, and controlled axial movement at the fracture site was prevented, resulting in nonunion of the fracture.

The femoral entry site can play a role in this complication. Intertrochanteric nails have an apex medial bend in the proximal aspect of the nail to allow the nail to easily traverse the intramedullary canal. The best point for introduction is at the tip of the greater trochanter. A slightly medial starting point is an acceptable alternative, but starting laterally on the greater trochanter invariably leads to a varus malreduction. In placing the nail, it is also important to establish the correct anterior-to-posterior position on the greater trochanter. A posterior starting point can cause an anterior direction of the nail, with consequent anterior cortex impingement of the distal tip of the nail. On the other hand, an anterior starting point may translate to the nail ending up more anterior in the distal femur, sliding on the anterior cortex. A slightly anterior starting point is also more favorable because it is easier to allow for anteversion of the femoral neck during cephalic screw placement. Even if a slightly anterior starting point is chosen, an external rotation of the nail guide to find the central placement of the cephalic screw into the anteverted femoral head may rotate the nail so that the radius of curvature of the implant is no longer in line with the bow of the femur. This could cause further impingement of the distal nail tip against the anterior cortex [19]. This type of scenario may be worsened if a medium-length cephalomedullary nail is chosen, because the distal tip of the nail across the apex of the anterior femoral bow will impinge on the distal return bend of the anterior cortex. In fact, at the apex of the femoral bow, the sliding of the distal tip of the nail on the anterior femoral cortex may jam due to the change of the femoral bow from anterior to posterior, as observed in our case report. When the surgeon uses a medium-length nail, he must be sure that there is no risk of distal jamming. If there is any doubt, it is better to plan a surgical dynamization removing the distal screw.

The latest generation of trochanteric nails offers the possibility of choosing between different lengths of nail to implant and different distal locking configurations to promote healing of the fracture. Due to femoral anterior shaft bowing, anterior cortical impingement of the distal tip of the nail may prevent sliding of the nail within the diaphyseal canal. As a result, dynamic distal locking with an elliptical hole can be ineffective if movement of the distal nail segment within the diaphyseal canal is hindered. A medium-length trochanteric nail placed with the tip of the nail near the apex of the antecurvation may predispose to anterior intramedullary nail impingement.

For these reasons, the surgeon must be aware of patients with excessive curvature. Caution is recommended in the use of medium-length trochanteric nails for unstable trochanteric fractures, in order to avoid compromising dynamic distal locking. Distal jamming of the nail may in fact compromise dynamic compression at the fracture, resulting in nonunion and breakage of the osteosynthesis device.

## References

[1] Lichtblau S (2008). The instable intertrochanteric hip fracture. Orthopedics.

[2] Kaplan K, Miyamoto R, Levine BR et al (2008) Surgical management of hip fractures: an evidence-based review of the literature. II. Intertrochanteric fractures. J Am Acad Orthop Surg 16(11):665–673 10.5435/00124635-200811000-00007 18978289

[3] Rebuzzi E, Pannone A, Schiavetti S (2002). IMHS clinical experience in the treatment of peritrochanteric fractures. The result of a multicentric Italian study of 981 cases. Injury.

[4] Kyle RF, Gustilo RB, Premer RF (1979). Analysis of six hundred and twenty-two intertrochanteric hip fractures. J Bone Joint Surg.

[5] Steinberg GG, Desai SS, Kornwitz NA (1988). The intertrochanteric hip fracture: a retrospective analysis. Orthopedics.

[6] Sommers MB (2004). A laboratory model to evaluate cutout resistance of implants for pertrochanteric fracture fixation. J Orthop Trauma.

[7] Jones HW, Johnston P, Parker M (2006) Are short femoral nails superior to the sliding hip screw? A meta-analysis of 24 studies involving 3,279 fractures. Int Orthop 30(2):69–78 10.1007/s00264-005-0028-0 PMC2532072 16496147

[8] Werner-Tutschku W (2002). Intra- and perioperative complications in the stabilization of per- and subtrochanteric femoral fractures by means of PFN. Unfallchirurg.

[9] Grisso JA, Kelsey JL, Strom BL (1991). Risk factors for falls as a cause of hip fracture in women. The Northeast Hip Fracture Study Group. N Engl J Med.

[10] Marsh JL, Slongo TF, Agel J, Broderick JS, Creevey W, DeCoster TA, Prokuski L, Sirkin MS, Ziran B, Henley B, Audigé L (2007) Fracture and dislocation classification compendium: Orthopaedic Trauma Association Classification, Database and Outcomes Committee. J Orthop Trauma 21(Suppl 10):S1–S163 10.1097/00005131-200711101-00001 18277234

[11] Egol KA, Chang EY, Cvitkovic J (2004). Mismatch of current intramedullary nails with the anterior bow of the femur. J Orthop Trauma.

[12] Ostrum RF, Levy MS (2005). Penetration of the distal femoral anterior cortex during intramedullary nailing for subtrochanteric fractures: a report of three cases. J Orthop Trauma.

[13] Kregor PJ, Obremskey WT, Kreder HJ (2005). Unstable pertrochanteric femoral fractures. J Orthop Trauma.

[14] Leung KS, Procter P, Robioneck B et al (1996) Geometric mismatch of the Gamma nail to the Chinese femur. Clin Orthop 323:42–48 10.1097/00003086-199602000-00006 8625605

[15] Harma A, Germen B, Karakas HM (2005). The comparison of femoral curves and curves of contemporary intramedullary nails. Surg Radiol Anat.

[16] Bong MR, Kummer FJ, Koval KJ (2007). Intramedullary nailing of the lower extremity: biomechanics and biology. J Am Acad Orthop Surg.

[17] Hwang JH, Oh JK, Han SH (2008). Mismatch between PFNa and medullary canal causing difficulty in nailing of the pertrochanteric fractures. Arch Orthop Trauma Surg.

[18] Menezes DF, Gamulin A, Noesberger B (2005). Is the proximal femoral nail a suitable implant for treatment of all trochanteric fractures?. Clin Orthop.

[19] Lundy DW (2007). Subtrochanteric femoral fractures. J Am Acad Orthop Surg.

[20] Koval KJ, Pollak AN, Winquist RA (2011) Intramedullary nailing of the femur (instructional course). In: AAOS Annual Meeting, San Diego, CA, USA, 15–19 Feb 2011, pp 12–18

[21] Caiaffa V, De Vita D, Laforgia R (2007). Treatment of peritrochanteric fractures with the Endovis BA cephalomedullary nail: multicenter study of 1091 patients. J Orthopaed Traumatol.

[22] Makridis KG, Georgaklis V, Georgoussis M (2010). Comparing two intramedullary devices for treating trochanteric fractures: a prospective study. J Orthop Surg Res.

[23] Apel DM, Patwardhan A, Pinzur MS (1989). Axial loading studies of unstable intertrochanteric fractures of the femur. Clin Orthop.

[24] Maniscalco P, Bertone C, Rivera F (2001). Use of a modified IMHS for unstable intertrochanteric fractures. J Orthopaed Traumatol.

